# Pathogenicity of Highly Pathogenic Avian Influenza Virus H5N1 in Naturally Infected Poultry in Egypt

**DOI:** 10.1371/journal.pone.0120061

**Published:** 2015-05-11

**Authors:** Ibrahim Thabet Hagag, Shimaa M. G. Mansour, Zerui Zhang, Ahmed A. H. Ali, El-Bakry M. Ismaiel, Ali A. Salama, Carol J. Cardona, James Collins, Zheng Xing

**Affiliations:** 1 Department of Virology, Faculty of Veterinary Medicine, Zagazig University, Zagazig, Egypt; 2 Department of Veterinary and Biomedical Sciences, College of Veterinary Medicine, University of Minnesota, Twin Cities, Saint Paul, Minnesota, United States of America; 3 Veterinary Diagnostic Laboratory, College of Veterinary Medicine, University of Minnesota, Twin Cities, Saint Paul, Minnesota, United States of America; 4 The State Key Laboratory of Pharmaceutical Biotechnology and Medical School, Nanjing University, Nanjing, China; Erasmus Medical Center, NETHERLANDS

## Abstract

Highly pathogenic avian influenza virus (HPAIV) H5N1 has been endemic in Egypt since 2006, and there is increasing concern for its potential to become highly transmissible among humans. Infection by HPAIV H5N1 has been described in experimentally challenged birds. However, the pathogenicity of the H5N1 isolated in Egypt has never been reported in naturally infected chickens and ducks. Here we report a 2013 outbreak of HPAIV H5N1 in commercial poultry farms and backyards in Sharkia Province, Egypt. The main symptoms were ecchymosis on the shanks and feet, cyanosis of the comb and wattles, subcutaneous edema of the head and neck for chickens, and nervous signs (torticollis) for ducks. Within 48-72 hrs of the onset of illness, the average mortality rates were 22.8-30% and 28.5-40% in vaccinated chickens and non-vaccinated ducks, respectively. Tissue samples of chickens and ducks were collected for analyses with cross-section immunohistochemistry and real-time RT-PCR for specific viral RNA transcripts. While viral RNA was detected in nearly all tissues and sera collected, viral nucleoprotein was detected almost ubiquitously in all tissues, including testis. Interestingly, viral antigen was also observed in endothelial cells of most organs in chickens, and clearly detected in the trachea and brain in particular. Viral nucleoprotein was also detected in mononuclear cells of various organs, especially pulmonary tissue. We performed phylogenetic analyses and compared the genomic sequences of the hemagglutinin (HA) and nonstructural proteins (NS) among the isolated viruses, the HPAIV circulated in Egypt in the past and currently, and some available vaccine strains. Further analysis of deduced amino acids of both HA and NS1 revealed that our isolates carried molecular determinants of HPAIV, including the multibasic amino acids (PQGERRRK/KR*GLF) in the cleavage site in HA and glutamate at position 92 (D92E) in NS1. This is the first report of the pathogenicity of the HPAIVH5N1 strain currently circulating in naturally infected poultry in Egypt, which may provide unique insights into the viral pathogenesis in HPAIV-infected chickens and ducks.

## Introduction

Highly pathogenic avian influenza virus (HPAIV) H5N1 persists in natural reservoirs of aquatic birds and waterfowl. In 1996 it was discovered that infected wild geese in Southern China were responsible for transmitting the virus to poultry [1]. In 2003, HPAIV H5N1 began to spread from Southeast Asia to other regions, and in the past decade it has caused numerous outbreaks on poultry farms in over 60 countries in Asia, Africa, and Europe. While the virus remains highly pathogenic to poultry and humans, it has also evolved to become pathogenic to domestic ducks beginning in 2002, and has killed wild waterfowl beginning with the outbreaks in Qinghai Lake in 2005 [2–5]. HPAIV H5N1 has caused severe agricultural and economic burdens with hundreds of millions of poultry culled, and poses a serious public health threat with over 370 human deaths reported since 2003 [6, 7].

HPAIV H5N1 was first transmitted to Africa in 2006 and spread quickly, with outbreaks reported in Nigeria, Egypt, Cameroon, and other African countries [8]. Notably, since then it has become endemic only in Egypt, which is the largest poultry producing country of the Arab nations [9], with regularly reported outbreaks, enormous economic losses in poultry industry, and confirmed human cases with high mortality [10]. While breeders' farms may have strict biosecurity, most commercial farms and household backyards, which comprise the majority of poultry producers in the country, practice poor biosecurity measures [11]. In addition, Egyptian customers rely heavily on live bird markets, where birds of different species and ages and from various locations are offered for trade and slaughter, providing numerous opportunities for viral transmission [12]. Since early control plans including culling infected birds, implementing quarantine measures, and movement restrictions have failed to contain the virus since its emergence in early 2006, control strategies have shifted to massive vaccination programs with inactivated H5N1 or H5N2 viruses, virus surveillance in poultry sectors, and preemptive culling of infected birds [11–13]. These new strategies have significantly reduced outbreaks in recent years. However, surveillance data reveal continuous and wide circulation of the virus in vaccinated and non-vaccinated commercial farms, household backyards, and live bird markets [14]. In addition, the virus may be undergoing genetic divergence, evolving into multiple genotypes. Remarkably, the H5N1 isolates circulating in Egypt that originated from the Qinghai Lake H5N1 viruses possess several critical genetic hallmarks, including the mutations in HA154-156, where a glycosylation site is missing, and PB2627K [15, 16]. The first renders the virus capable of transmission in mammals, and the second increases viral replication in humans, causing concern that HPAIV H5N1 variants may emerge in Egypt with increased potential for transmissibility in mammals [17].

The HPAIV H5N1 viruses circulating in Egypt since 2006 are highly pathogenic to chickens and can be lethal to ducks [12]. Since becoming endemic in Egypt, the virus has spread from farm to farm, even under immune pressure produced from vaccinated H5 viruses, which may lead to its evolution and mutation [14]. There is limited understanding of the viral pathogenicity and pathogenesis of the current Egyptian H5N1 viruses, the most common cause of outbreaks in poultry in the country.

In this study we report a recent outbreak of the HPAIV H5N1 in commercial poultry farms and backyards in Sharkia Province, Egypt. Our data indicate that the mortality rates were 22.8–30% in broilers and layers that were vaccinated once with inactivated H5 vaccines, in contrast to the mortality rate of 0.14% in layers which were vaccinated twice. The mortality rates for two backyard flocks of ducks were 28.5 and 40%, respectively, and none of the ducks were vaccinated. Using immunohistochemistry staining we detected viral nucleoprotein in multiple organs and tissues, including trachea, lung, brain, spleen, pancreas, liver, proventriculus, bursa of Fabricius, and testis. The resultant HPAI H5N1 viruses A/chicken/Egypt/IT-1/2013, A/chicken/Egypt/IT-2/2013, and A/duck/Egypt/IT-3/2013 were isolated, and their HA and NS genes analyzed by comparing with the H5N1 viruses previously isolated in Egypt, neighboring countries and worldwide.

## Materials and Methods

### Viruses

HPAIV H5N1 strains were isolated from chicken and duck tissue samples in commercial poultry farms or backyards in Sharkia Province, Egypt, from February to May 2013. Tissue samples were collected and ground in DMEM cultural medium and centrifuged at 500xg for 10 min. The supernatants were collected and inoculated into 9- to 11-old embryonated chicken eggs, which were then incubated at 37°C. Allantoic fluids were collected and tested with a standard hemagglutination (HA) assay. For HA positive samples, viral RNA was extracted and viral subtypes were identified by reverse transcriptase-polymerase chain reaction (RT-PCR) using a set of subtype-specific primers for viral genes (Table 1), and then further confirmed using nucleotide sequencing. The viruses were aliquoted and stored at -80°C. All animal work was performed with accomplice to the rules and approved ahead by the ethics committee for animal studies at Zagazig University, Egypt.

**Table 1:** Sequences of the Oligonucleotide Primers Used in the Study.

Target Gene	Primer Forward (5’>3’)	Primer Reverse (5’>3’)	Product Size (bp)	References
**H5**	**H5-kha-1**	**H5-kha-3**	~317	**[18]**
	CCTCCAGARTATGCMTAYAAAATTGTC	TACCAACCGTCTACCATKCCYTG		
**N1**	**N1-54F**	**N1-298R**	~245	**[19]**
	TCARTCTGYATGRYAAYTGG	GGRCARAGAGAKGAATTGCC		
**NS**	**Bm-NS-1**	**Bm-NS-890R**	890+29	**[20]**
	TATTCGTCTCAGGGAGCAAAAGCAGGGTG	ATATCGTCTCGTATTAGTAGAAACAAGGGTGTTTT		
**M**	**M+25F**	**M-124R**	**-**	**[22]**
	AGATGAGTCTTCTAACCGAGGTCG	TGCAAAAACATCTTC AAGTCTCTG		
**M Gene Probe**	**M+64probe**		**-**	**[22]**
	FAM-TCAGGCCCCCTCAAAGCCGA-TAMRA			

### RNA Extraction, RT-PCR, and Realtime RT-PCR

**Fig 1:**
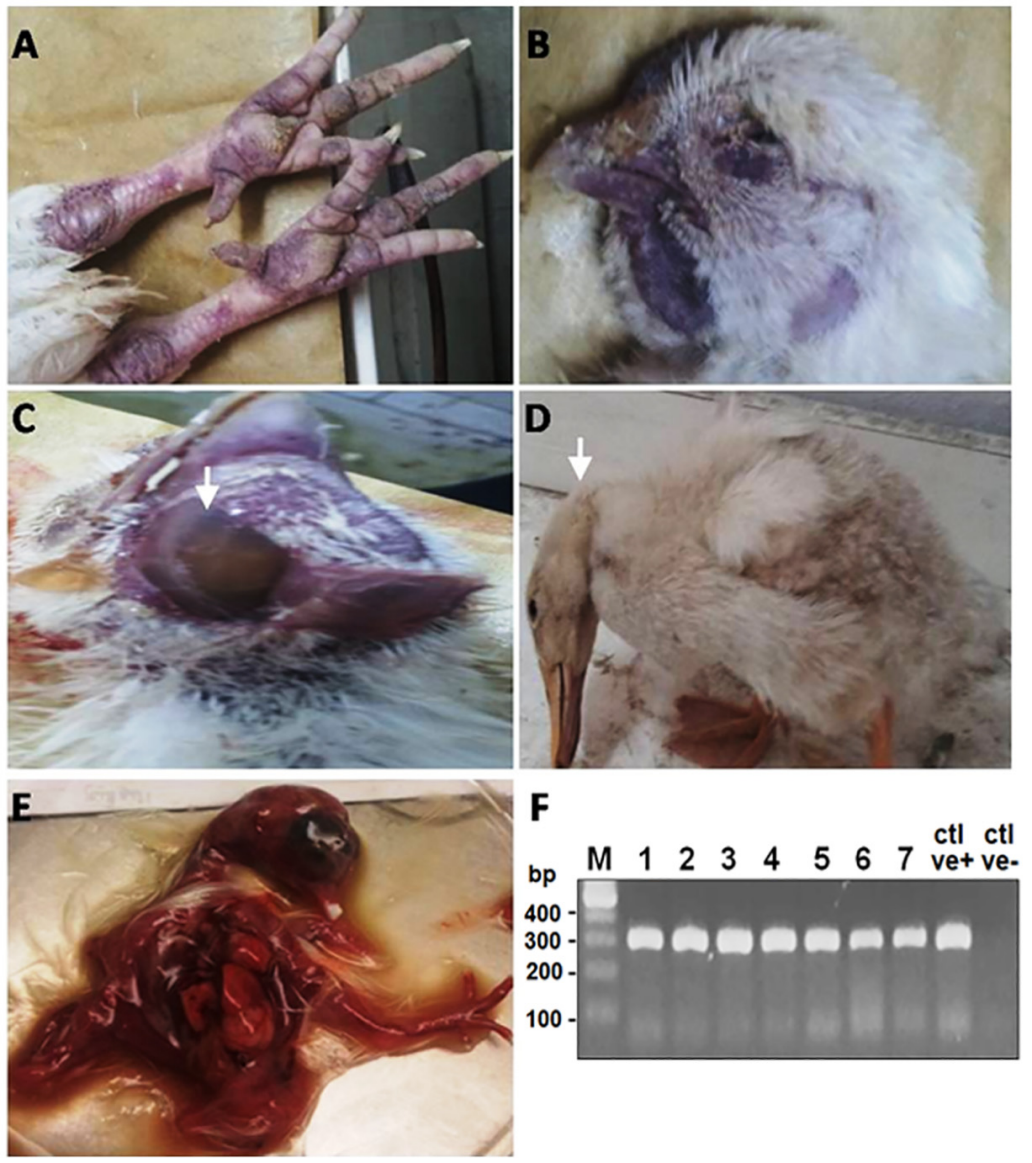
Naturally infected chickens and ducks by HPAIV H5N1. (**A**) Chickens: subcutaneous hemorrhages in non-feathered skin. **(B**) Chickens: cyanosis of comb & wattles. (**C**) Chickens: facial edema. (**D**) Ducks: nervous signs, torticollis. (**E**) Infected chicken embryos: chicken embryos were inoculated with supernatants of homogenized testicular tissue of naturally infected layer chickens; the inoculated embryos died within 48hrs with severe congestion and hemorrhages after the first passage. (**F**) PCR amplification for HA gene showing bands of 317bp in size. First lane: Molecular marker of 100 bp, Lane 1–7: Positive samples, Ctrl +ve: Positive AIV subtype H5N1, Ctrl-ve: Negative control.

Viral RNA was extracted from allantoic fluids with Blood/Liquid Sample Total RNA Rapid Extraction Kit (Bioteke Corporation, China) following manufacturer’s instructions. Approximately 1 μg of RNA (5 μl) was reverse transcribed to cDNA using cDNA DiaStarRT Kit (Solgent Co., Korea) according to the manufacturer’s protocol. The cDNA were used as templates for PCR with specific primers to H5 and N1 [18, 19], which was carried out for 35 cycles. PCR amplicons were subjected to electrophoresis in a 1.5% agarose gel, and fragments of H5 subtype HA and N1 subtype neuraminidase (NA) genes of expected sizes were detected (Fig 1F).

For realtime RT-PCR, total RNA were prepared from various tissue and serum samples and reverse transcribed to cDNA. TaqMan-based realtime RT-PCR (Applied Biosystems, USA) was performed with 1 μl of cDNA in a volume of 25 μl to measure viral M gene transcripts in the tissues using a set of M gene specific primers and probe (Table 1). The probe was labeled with 5’-FAM and 3’-TAMRA. The reaction was run with ABI 7500 and each assay was repeated three times with positive and negative controls included with the tested samples.

### Viral Gene Cloning, Sequencing, and Phylogenetic Analysis

Viral RNA extracted from the allontoic fluids was reverse transcribed with Superscript III Reverse Transcriptase (Invitrogen, USA) and a Uni-12 primer [20]. The cDNA was amplified by PCR with Taq DNA polymerase (Takara, Japan) and primers specific for influenza virus gene segment [20]. PCR products were cloned into a TA cloning vector (Invitrogen, USA) and the nucleotide sequences determined by DNA sequencing.

Phylogenetic analyses of the HA and NS gene were based on nucleotides 791–1100 (309 bases) of HA and 39–704 (666 bases) of NS. All gene sequence data of known H5N1 strains were collected from the NCBI Influenza Database. Multiple alignments were constructed using ClustalW Multiple Alignment with the MegAlign module of DNAStar software (Lasergene version 7.2, DNASTAR, Madison, WI, USA). The neighbor-joining method with Kimura two-parameter distances was used for constructing the phylogenetic tree using the Mega 5.0, and the tree was rooted to the A/goose/ Guangdong/1/1996 virus sequence. The reliability of the internal branches was assessed by the p-distance substitution model and 1000 bootstrap replications. The HA and NS genotypes was determined using the Influenza A Virus FluGenome web server, (http://www.flugenome.org/) [21].

### Immunohistochemistry

Freshly collected tissues were fixed by 10% formalin and embedded in paraffin. For histopathology, cross-sections were prepared at 5 μm and stained with hematoxylin and eosin (HE). For immunohistochemistry (IHC) staining, a duplicate 4-μm section was cut, mounted on positively charged SuperFrost Plus microscope slides, dewaxed, and rehydrated. Antigen retrieval was performed by pressure-cooking for 25 mins in pH 6 citrate buffer. Endogenous peroxidases were blocked using 3% H_2_O_2_ for 15 mins. Non-specific bindings were blocked using normal goat serum at 1:10 in TBS for 15 mins. Slides were incubated overnight at 4°C with primary rabbit anti-influenza A nucleoprotein (NP) polyclonal antibody (Catalog ID19382; LifeSpan Biosciences, WA) at 1:50 dilution. The EnVision AP (DAKO K1396, Carpinteria, CA) detection system and nuclear fast red (DAKO K1396) were used as chromogens. Sections were counterstained with Mayer’s hematoxylin. Positive and negative control sections were included in each IHC run. All tissues were systematically screened for microscopic lesions. The intensity of viral antigen staining in each section was also scored as following: (-), no antigen staining; (+), infrequent; (++), common; and (+++), widespread staining.

### Statistical Analysis

Data were collected and continuous variables were analyzed using one-way analysis of variance (*ANOVA*), then comparison of means was carried out with Duncan’s multiple range tests (*DMRT*) and summarized as mean ± standard deviation.

## Results

### Outbreaks of HPAIV H5N1 in commercial poultry farms and backyards

Beginning in February 2013, chickens and ducks in commercial poultry farms and backyards in Sharkia Province, Egypt, became sick with symptoms including ecchymosis on the shanks and feet, cyanosis of the comb and wattles, subcutaneous edema of head and neck in chickens, and nervous signs (torticollis) in ducks, characteristics of HPAIV infection, as shown in Fig 1A–1D. Birds began to die within 48–72 hrs of the onset of illness. The mortality rates for two unvaccinated backyard flocks of ducks consisting of 14 and 20 birds were 28.5 and 40%, respectively (Table 2). On the other hand, the mortality rates were 22.8 to 30% in broilers and layers that were vaccinated once with inactivated H5 vaccines, and 0.14% in layers that were vaccinated twice (Table 2). The flock sizes of the commercial chicken farms ranged from 3,900 to 5,000 for both broilers and layers.

**Table 2:** The Outbreaks of HPAIV H5N1 in the Egyptian Commercial Poultry Farms and the Virus Identification.

Flock No.	Species	Rearing System/Breed	Age/ Days	Clinical Characteristics	Average Mortality Rates	No. of Sampled Birds	No. of H5N1 +ve Birds	Results of HPAI H5N1 Infected Flocks			
								Viral Isolation	Routine HA	RT-PCR	IHC
**1**	Ducks	Backyard/ Mallard	25	Greenish diarrhea and nervous signs	28.50%	1	1	+ve	+ve	+ve	-ve
**2**	Ducks	Backyard/ Muscovy	30	(Torticollis)	40.00%	3	3	+ve	+ve	+ve	N/A
**3**	Chicken broilers	Commercial/ Cobb 500*	32		22.85%	9	9	+ve	+ve	+ve	+ve
**4**	Chicken broilers	Commercial/ Cobb 500*	27	Respiratory signs.	30.32%	9	6	+ve	+ve	+ve	+ve
**5**	Chicken broilers	Commercial/ Cobb 500*	35	Cyanosis of the head, comb and wattles Subcutaneous edema of head and neck with ecchymoses on the shanks and feet	24.19%	9	3	+ve	+ve	+ve	N/A
**6**	Chicken layers	Commercial/ Hyline*	105		24.50%	9	9	+ve	+ve	+ve	+ve
**7**	Chicken layers	Commercial/ Hyline**	101		0.14%	6	3	+ve	+ve	+ve	+ve

**Total**	Ducks	-	-	-	-	4	4	**-**	**-**	**-**	**-**
	Chickens	-	-	-	-	42	30	**-**	**-**	**-**	**-**

* Chicken broiler and layer flocks were vaccinated once with H5 inactivated vaccine.

** This chicken layer flock was vaccinated two times with H5 inactivated vaccine.

Backyard ducks were not vaccinated.

N/A: Not applied

+ve: Positive

Nine- to11-day-old chicken embryonated eggs were inoculated with samples from ground pulmonary, tracheal, and testicular tissues of dying or dead chickens or ducks. Embryos with extensive hemorrhage died within 48 hrs post inoculation. Severely congested embryos were observed in eggs inoculated with testicular tissues after the first passage (Fig 1E). Allantoic fluids were tested for hemagglutination (HA) reactivity with a routine HA assay and positive titers were detected. Further viral RNA was prepared from the allantoic fluids, which was reverse transcribed to cDNA for amplification of viral HA and NA fragments with polymerase chain reaction (PCR) using primers specific for theH5N1 HA and NA genes (Table 1). Results indicated that the isolates wereHPAIVH5N1 positive. The PCR results showed specific bands in 1.5% agarose gel at 317(Fig 1F) and 245 base pairs in size (data not shown) for HA and NA, respectively. HPAIV subtype H5N1 was detected in34 of 46 birds including both ducks and chickens (Table 2) with a detection rate of73.9%, including 30 of 42 (71.4%) chickens and 4 of 4 (100%) ducks, respectively (Table 2). Further nucleotide sequencing of the purified PCR products confirmed the isolates to be of the HPAIVH5N1 subtypes, and three viral isolates were designated as A/chicken/Egypt/IT-1/2013, A/chicken/Egypt/IT-2/2013, and A/duck/Egypt/IT-3/2013. The relevant nucleotide sequences were deposited at the NCBI GenBank database with deposition numbersKJ192204, KJ192205, KJ192206,KP311329, and KP311330 for the three viral strains, respectively.

### Viral RNA in chicken and duck tissues

Chicken and duck tissues including trachea, lung, liver, spleen, intestine, brain, testis, and serum were taken from sick or dead birds for total RNA preparation. After reverse transcription, cDNA of the samples were subjected to TaqMan-based semi-quantitative realtime RT-PCR (qRT-PCR) [22]with primers specific to the M gene to examine viral RNA in each organ type.

**Fig 2:**
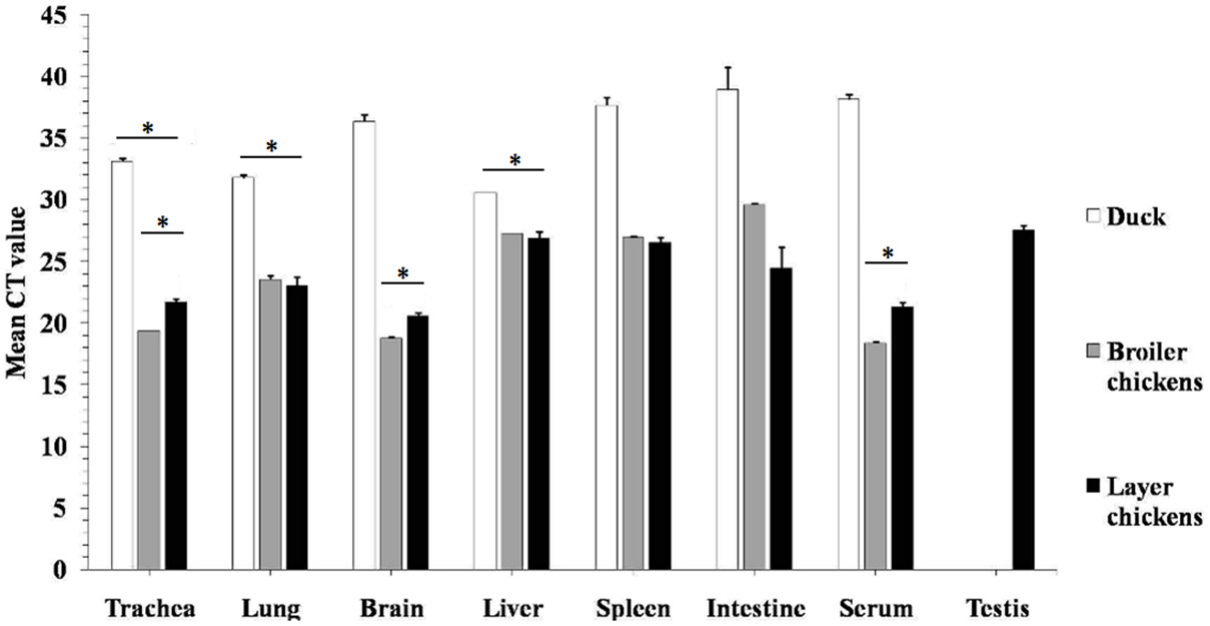
Detection of M gene transcripts by RT-PCR. Results were expressed as *C*t values; *C*t values lower than (35) were considered positive; *C*t values between 15 and 25 were considered strong positive, while those between 25 and 30 were considered moderate positive; *C*t values between 30 and 35 were considered weak positive. The tests were performed in triplicates for each sample and the C*t* numbers are average with 3xSD (* P<0.05).

As shown in Fig 2, viral RNA of the HPAIV H5N1 M gene was detected in all tissues tested both in chickens and ducks with the exception of testis, which was only positive for chicken layers. Higher levels of viral RNA were in general detected in tissues of broiler and layer chickens, including trachea, lung, spleen, intestines, brain, and serum, than in those of ducks (Fig 2). However, these samples from different birds cannot be compared because they came from natural outbreaks with uncertain timing of the course of infection. In broiler chickens, however, higher viral RNA levels appeared in brain, trachea, and serum samples. No significant differences in viral RNA levels were observed in various tissues of ducks, except for higher levels detected in trachea, lung, and liver tissues (Fig 2).

### Viral nucleoprotein detected in organs of chickens

Organs and tissues from sick or dead chickens from farms where outbreaks of HPAIV H5N1 occurred were prepared for cross-section slides with routine paraformaldehyde fixation, and subjected to IHC. An antibody to HPAI H5N1 nucleoprotein (NP) was used to stain the samples for detection of viral NP in tissues of infected birds.

**Fig 3:**
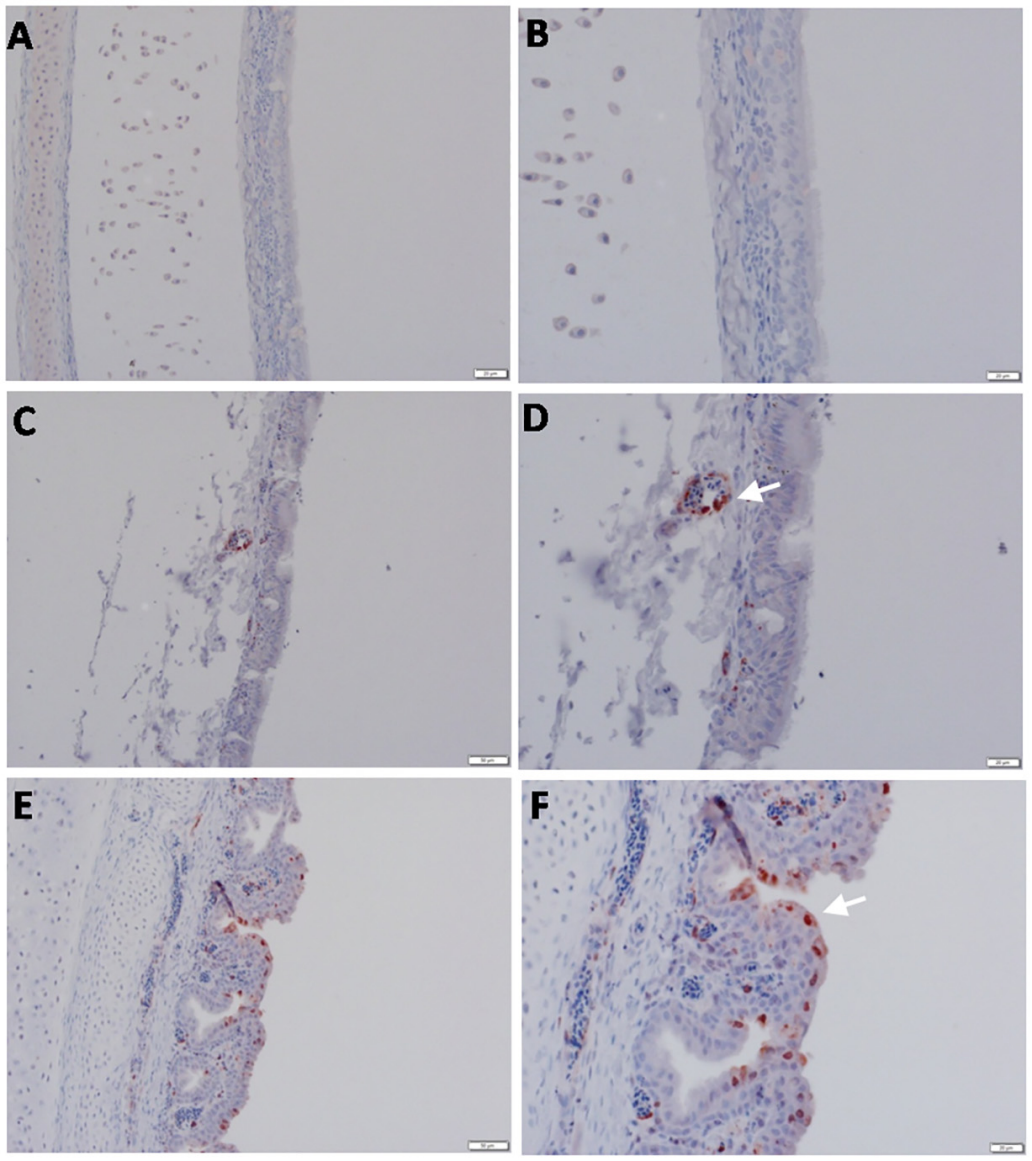
Detection of viral NP antigen in trachea by IHC. (**A** and **B**) Trachea of non-infected birds (X = 50 and 20μm, respectively). (**C** and **D**) Endothelial cells of trachea of infected birds (X = 50 and 20μm, respectively). **(E** and **F)** Epithelial cells of trachea of infected birds (X = 50 and 20μm, respectively).

**Fig 4:**
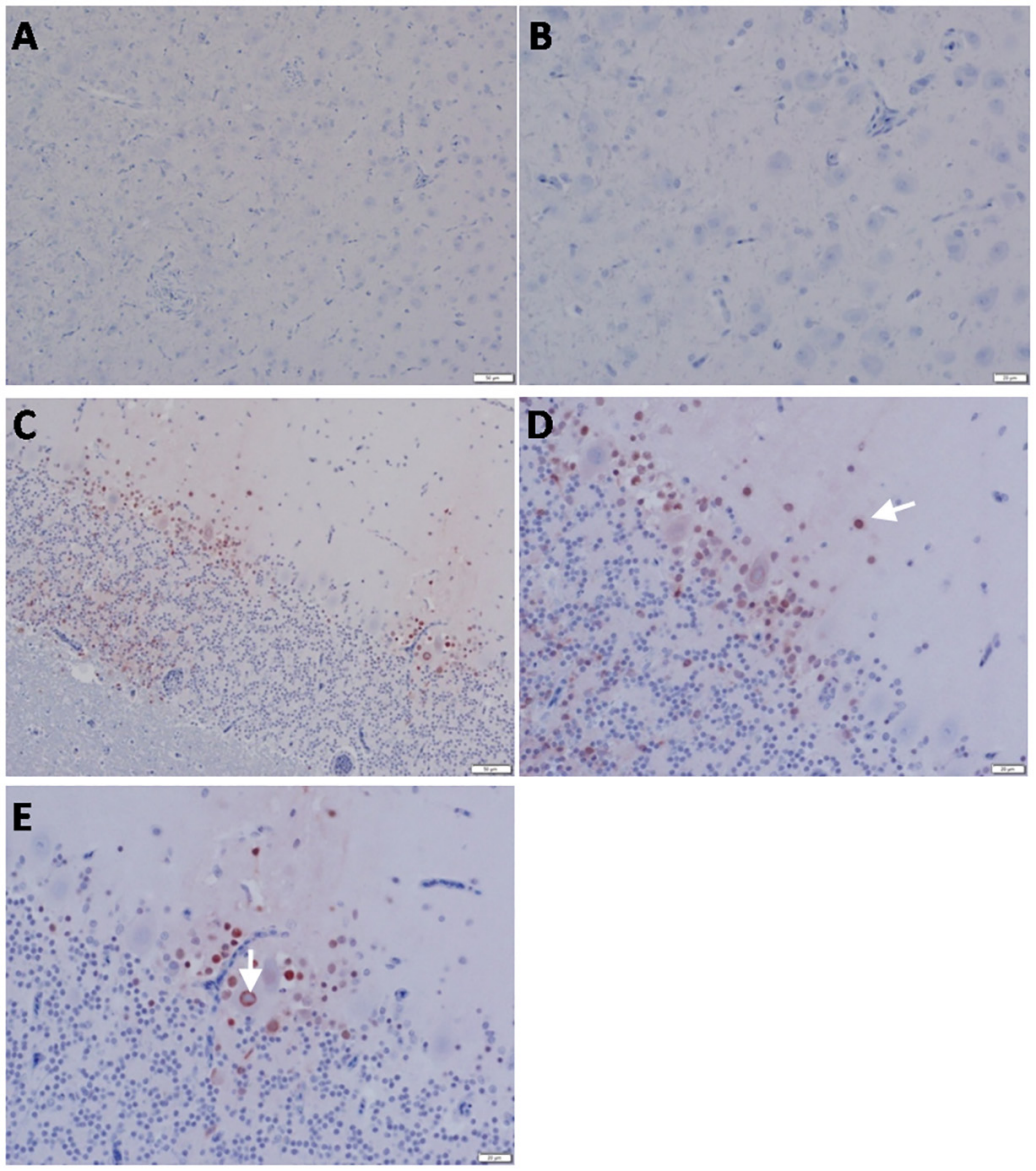
Detection of viral NP antigen in brain by IHC. (**A** and **B**) Brain of non-infected birds (X = 50 and 20μm, respectively). **(C**) Perkinji cell layer of cerebellum of infected birds (X = 50μm). (**D**) Neurons and glial cells of perkinji cell layer of cerebellum of infected birds (arrow) (X = 20μm). (**E**) Endothelial cells of perkinji cell layer of cerebellum of infected birds (arrow) (X = 20μm).

**Fig 5:**
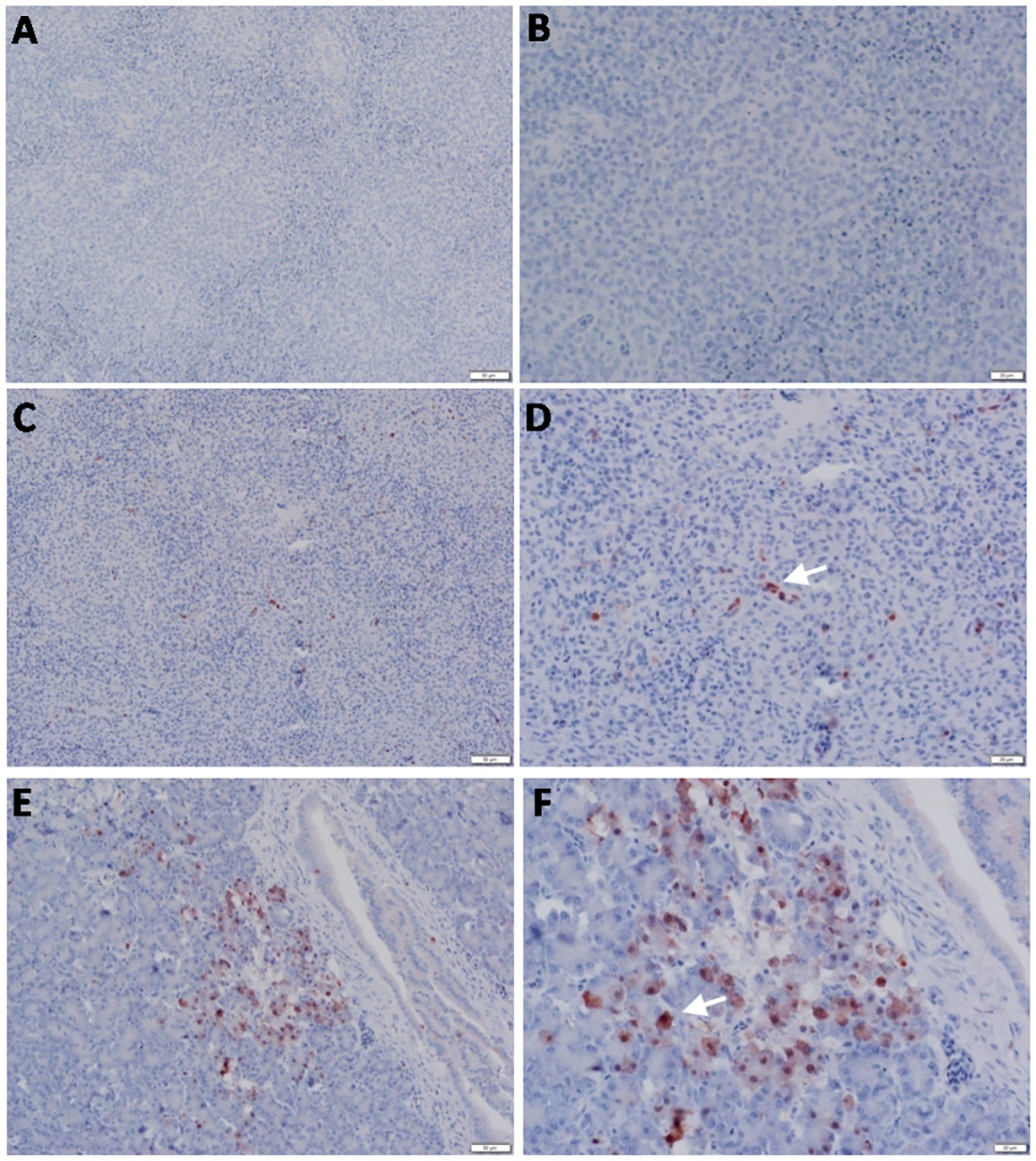
Detection of viral NP antigen in spleen and pancreas by IHC. **(A** and **B**) Spleen of non-infected birds (X = 50 and 20μm, respectively). **(C**) Lymphocytes in spleen of infected birds (X = 50μm). (**D**) Lymphocytes and mononuclear cells in spleen of infected birds (arrow) (X = 20μm). (**E** and **F)** Acinar epithelium of pancreas of infected birds (X = 50 and 20μm, respectively).

**Fig 6:**
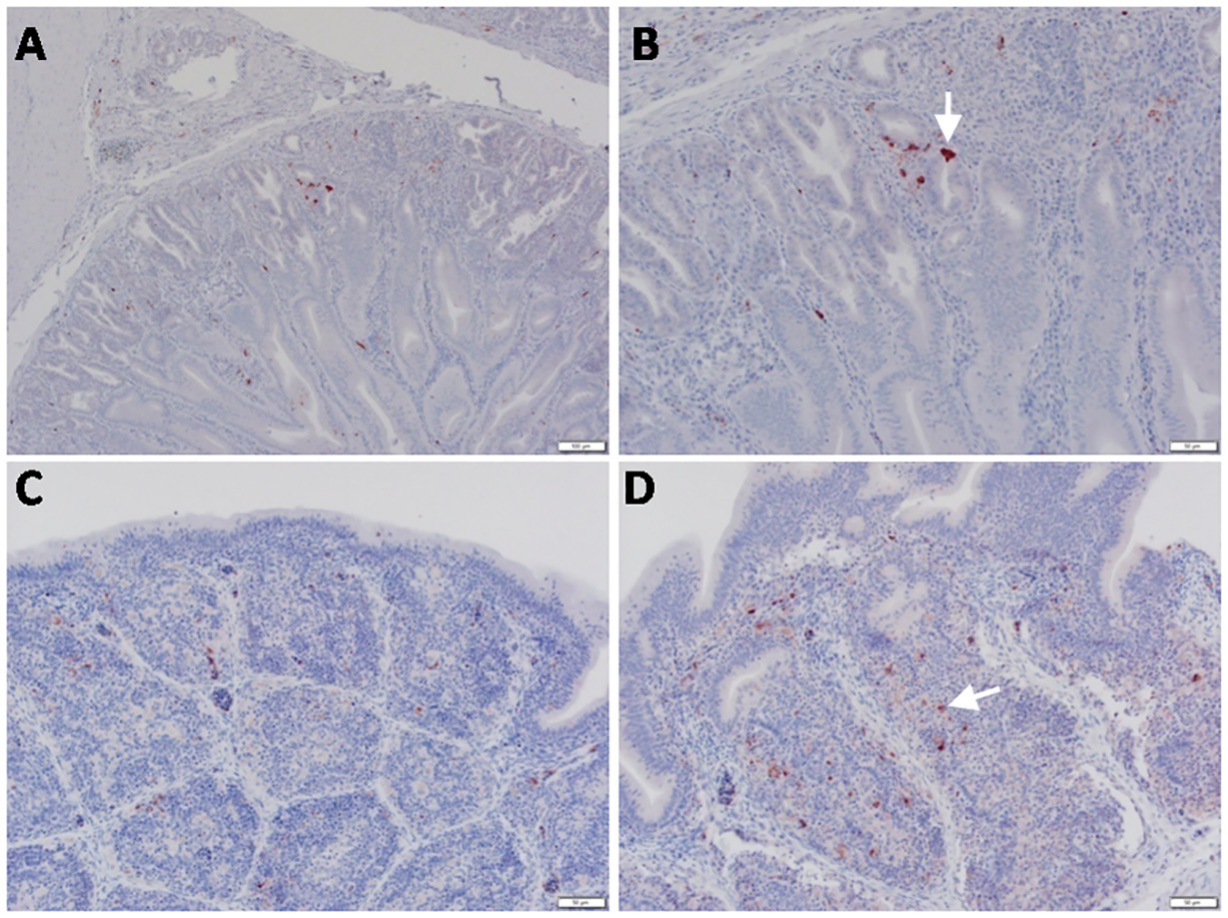
Detection of viral NP antigen in proventriculus and bursa of Fabricius by IHC. (**A**) Proventriculus of infected birds (X = 50μm). (**B**) Glandular epithelium of proventriculus (arrow) (X = 20μm). (**C** and **D**) Lymphocytes in follicular layer of bursa of Fabricius of infected birds (arrow) (X = 50 and 20μm, respectively).

**Fig 7:**
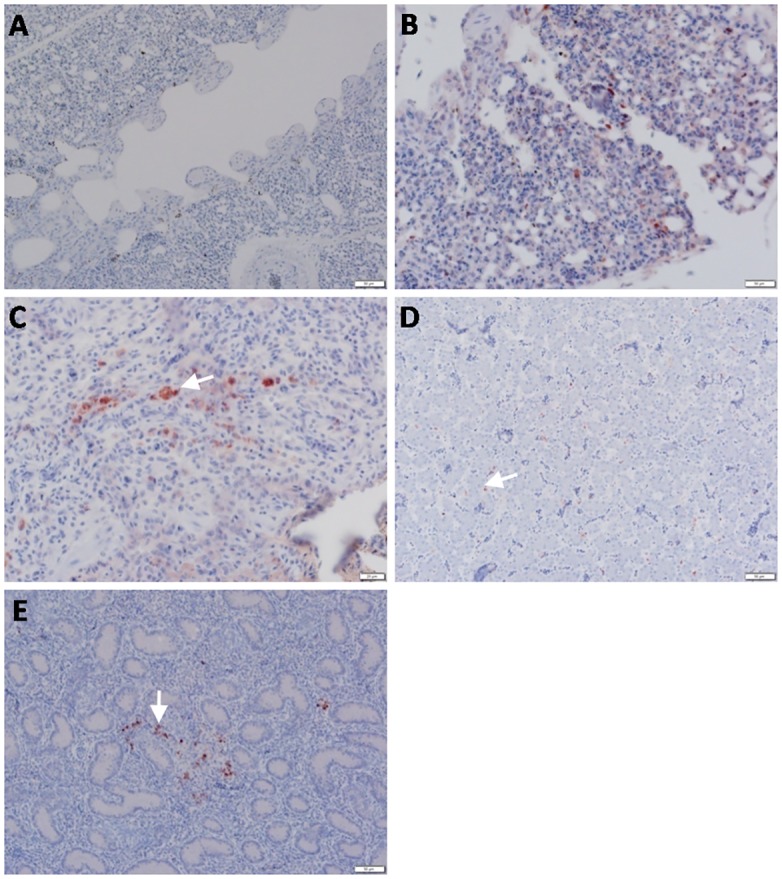
Detection of viral NP antigen in lung, liver and testisby IHC. (**A**) Lung of non-infected birds (X = 50μm). (**B** and **C**) Macrophages and mononuclear cells in lungs of infected birds (arrow) (X = 50 and 20μm, respectively). (**D**) Liver sinusoids of infected birds (inside van Kuppfer cells) (arrow) (X = 50μm). (**E**) Testis of infected birds (between seminephrous tubules of testicular tissue) (arrow) (X = 50μm).

As shown in Table 3 and Figs 3 through 7, viral NP was detected in all tested tissues including brain (Fig 4), pancreas and spleen (Fig 5), proventriculus and bursa of Fabricius (Fig 6), and lung and liver (Fig 7). Remarkably, the viral antigen was also detected in testicular tissue in between seminephrous tubules, even sticking to heads of sperm inside them (Fig 7E). The viral antigen was often associated with histologic lesions, although it was also observed in areas without detectable lesions.

**Table 3:** Summary of Virus Distribution in Tissues of the HPAIV H5N1-Infected Chickens IHC scoring system. (-) no viral antigen detected; (+) sporadic; (++) common; (+++) widespread.

Tissue	IHC Score	Type of Cells Expressing Virus Antigen
**Trachea**	+++	Endothelial cells and epithelial cells
**Lung**	+	Macrophages, Lymphocytes
**Brain**	+++	Endothelial cells, neurons and glial cells especially in perkinji cell layer
**Spleen**	+	Lymphocytes, Endothelial mononuclear cells
**Bursa**	+	Lymphocytes inside Follicular layer, endothelial cells
**Pancreas**	+++	Pancreatic acinar epithelium, macrophages and endothelial cells
**Proventriculus**	+	Glandular epithelium
**Testis**	++	Inter seminephrous space, intra seminephrous tubules sticking to sperms

**IHC scoring system:** (-) no viral antigen detected; (+) sporadic; (++) common; (+++) widespread.

Staining of most organs revealed a common feature characteristic of the viral antigen detected in their endothelial and mononuclear cells, which suggests that viral pathogenesis of the HPAIV H5N1 may be associated with endothelial invasion, and that the virus could be carried by infected monocytes. We identified viral NP in endothelial (Fig 3D) and epithelial (Fig 3F) cells of the trachea, neurons, glial cells of Perkinji cell layer (Fig 4C and 4D) and endothelial cells of brain (Fig 4E), lymphocytes and mononuclear cells of spleen (Fig 5D), acinar epithelium of pancreas (Fig 5F), glandular epithelium of proventriculus (Fig 6B), and lymphocytes of the follicular layer of the bursa (Fig 6D). A summary of viral antigen staining in various tissues examined is shown in Table 3.

We prepared samples from dead ducks and performed the same IHC staining on duck tissues as chicken tissues with the anti NP antibody. However, we failed to detect viral NP in all tissues examined (data not shown). Our failure could be attributed to the preparation of the samples, since they were collected from dead ducks in the field. Although viral RNA was still present and live virus isolated successfully (Figs 1 and 2), no sufficient viral antigens existed in the tissues due to prolonged exposure at ambient temperature.

### Sequence characterization of the HPAIV H5N1 HA and NS genes

**Fig 8:**
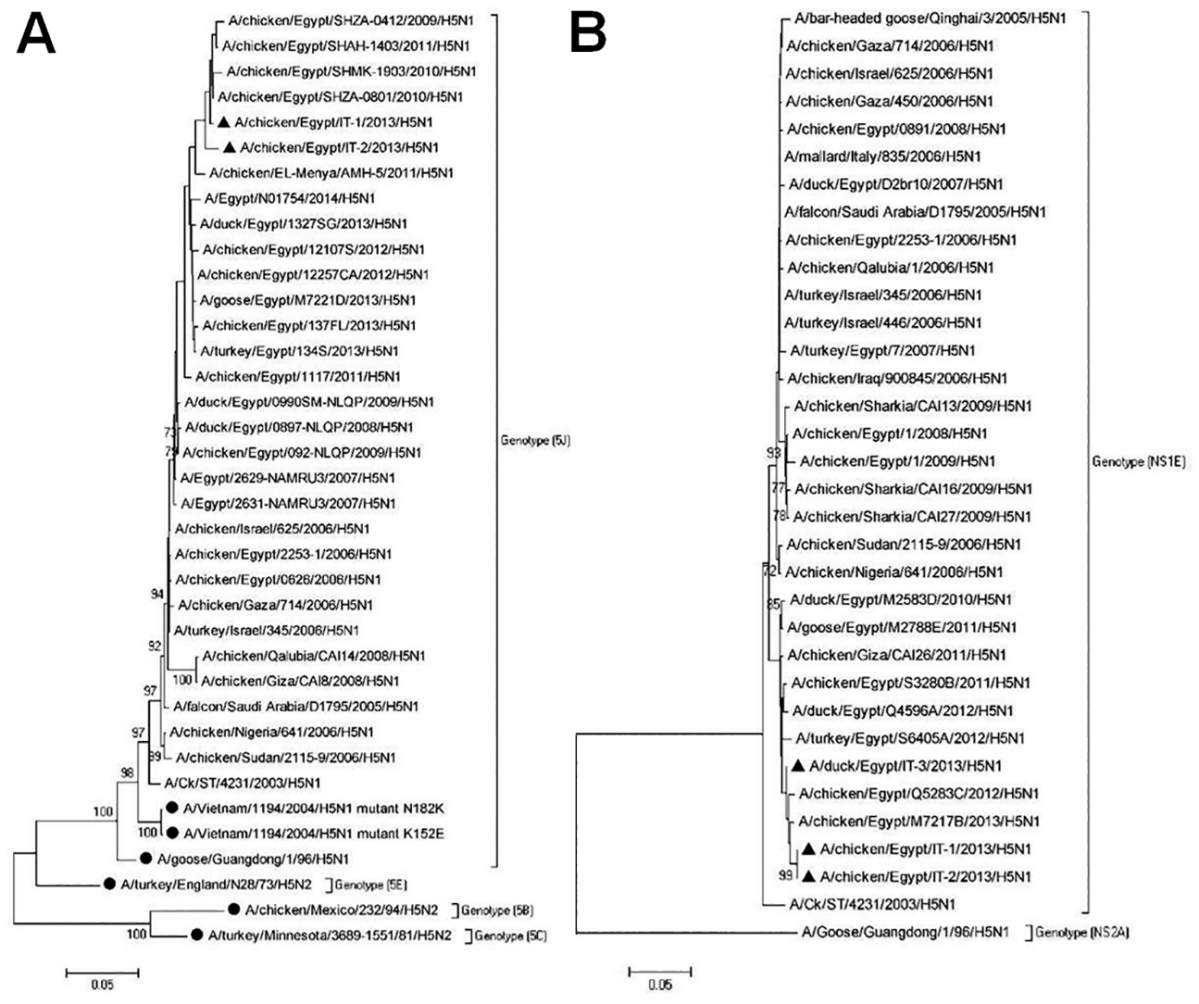
Phylogenetic analysis of nucleotide sequences of the HA and NS genes. **(A**) Phylogenetic analysis of HA gene nucleotide sequences at the cleavage site. The sequences of known HPAIV HA genes were obtained from GenBank. Vaccine H5 strains were included in the tree and marked with solid circles. The tree was constructed with multiple alignment of a 309 base-nucleotide sequence of HA genes using the Neighborhood-joining method in MEGA5. The tree topology was evaluated by 1,000 bootstrap analyses. The viruses isolated in this study are marked with solid triangle. (**B**) Phylogenetic analysis of NS gene on the basis of nucleotide sequences of the complete coding region (822 bases). The tree was constructed using Neighborhood joining method with bootstrap values calculated for 1,000 replicates and cut off value of 50%. Sequences of the isolates from this study are marked with solid triangles.

Genomic sequences of the HA genes from the isolated viruses were sequenced and the cleavage sites were compared with those of known H5N1 viruses. We found that the HA of our isolates encodes a multibasic amino acid motif, 321-PQGERRRK/KR*GLF-333, at the HA cleavage site, which is a characteristic feature of all HPAIV H5N1 strains. Interestingly, we found that one of our isolates has a substitution of amino acid (R325K) at this cleavage site, to make it PQGEKRRK/KR*GLF. A phylogenetic tree was constructed, indicating that the HA gene belongs to (5J) genotype (Fig 8A). Our isolates formed a uniform cluster, together with the HPAIVH5N1 viruses from Egypt isolated in 2009, 2010, 2011, 2012, and 2013; however, this cluster was not identical to the HPAIV H5 strains used for commercial vaccine development in Egypt, and was also phylogenetically distant from the viruses isolated from Egypt in 2006, 2007, and 2008.

We also sequenced the NS genomic segments, which are 822 nucleotides in length and encode for NS1 and NS2/NEP proteins. A phylogenetic tree was constructed indicating that the NS1 gene of our isolates belongs to the NS1EQinghai Lake-like genotype (Fig 8B). Our isolates also formed a uniform cluster, together with the HPAIVH5N1 viruses from Egypt isolated in 2010, 2011, 2012, and 2013; however, this cluster was phylogenetically distant from other HPAIV H5N1 isolated from Egypt in 2006, 2007, 2008, and 2009.

We found that the NS1 protein of our H5N1 viruses had a deletion of the ^80^TMASV^84^motif and was 225 amino acids in length. The NS1 had a glutamate at position 92 (92E) and its carboxyl terminus harbored the E-S-E-V motif, both of which have been identified as pathogenic determinants of highly pathogenic viruses [23].

## Discussion

In this report we demonstrated that the HPAIV H5N1 is still circulating in commercial poultry farms and backyards and causing outbreaks with significant mortality to both chickens and domestic ducks in Egypt. Our data showed that vaccination with H5N1 and H5N2vaccines may provide significant protection, especially to layers which were vaccinated twice. For both broilers and layers in commercial farms, there appears to be protection with even one-time vaccination when compared to a mortality of over 70% in non-vaccinated chickens [24]. Unfortunately, we did not perform serological test to evaluate the immunological status or vaccine efficacy in chickens of the commercial flocks, and therefore are unable to assess how well the mortality is correlated to the vaccination. Therefore, we cannot conclude emphatically that the much lower mortality in the commercial layers, which were vaccinated twice, was absolutely attributed to the boosted immunity.

Vaccine efficacy may best be assessed in the setting of natural outbreaks, which apparently differs from that designed experimentally. In naturally infected free-living birds, the clinical and pathologic manifestations of an HPAIV infection may be influenced by multiple factors including the age of the bird, the dosage of virus and routes of viral exposure, the presence of concomitant infections, and the levels of immunity acquired from vaccination or during previous exposure to influenza viruses [25, 26], which could be significantly different from trials with experimental challenges. Data about vaccine protection and efficacy from natural outbreaks may be more valuable when case studies are carefully planned and serological survey is thoroughly performed and assessed. Even though the mortalities were lowered, the pathogenicity of the infection appeared to be severe, and the virus was highly virulent in sick chickens as shown in Fig 1 and Table 2.

Pathogenicity of infection by HPAIV H5N1 has been studied experimentally in chickens and domestic ducks. Vietnam HPAIV H5N1 caused high mortality in two- and four-week-old SPF White Leghorn chickens (*G*. *gallusdomesticus*) (100%, 48/48) with mean death times (MDT) from 36 to 48 hrs, and two- and five-week-old Pekinwhite ducks (*Anasplatyrhynchus*) (98.4%, 63/64) with MDTs from 2.7 to 4.4 days [27]. Pathogenicity of the Egyptian HPAIV H5N1 have been tested experimentally only in domestic ducks [28]. While A/ck/Egypt/08 killed 8/8 (100%) with an MDT of 4.1 days, A/ck/Egypt/07 killed 4/8 (50%) with an MDT of 7 days, indicating that HPAIV H5N1 isolates differ in their virulence. Although these two isolates are considered to have evolved from the same origin [28], they are far apart within the clade 2.2 in the phylogenetic tree and have evolved different pathogenicity since the HPAIV H5N1 of clade 2.2 was introduced into Egypt. On the other hand, pathogenicity observed in these experimental challenges cannot be directly compared with that in natural outbreaks. Apparent differences exist between SPF birds with a certain age group infected experimentally by the intranasal route (IN) and poultry of various ages in commercial farms by natural exposure. The mortality rates in our report were lower in duck and even much lower in once- and twice-vaccinated chickens during the outbreaks. However, for sick birds the symptoms were severe. Systemic infection was observed and nervous disorders were obvious in both chickens and ducks, and infection was confirmed by viral antigen detection.

The current H5N1 viruses, A/chicken/Egypt/IT-1/2013 and A/chicken/Egypt/IT-2/2013, clearly demonstrate their pantropism in tissues and organs as virulent HPAIV strains. The viral RNA and NP antigen were detected in multiple tissues, including trachea, lung, brain, liver, spleen, pancreas, intestines, serum, proventriculus, bursa of Fabricius, and testis in infected chickens, similar to those observed in chickens infected with the Vietnamese H5N1 virus [28]. Neurotropism of the HPAI H5N1 virus has been previously described [29–32]. Our study shows that high levels of viral RNA were detected in the brains of infected chickens (Fig 2) and that viral NP antigen was observed in the nuclei of neurons and glial cells of the brain, clear signs that the virus replicates in brains. We could also conclude that even though vaccination may lower mortality rates, it does not change pantropism of HPAIV H5N1 in sick birds.

Different pathways have been proposed by which the HPAI H5N1 virus infects the central nervous system (CNS) in chickens. It has been hypothesized that the virus could reach the CNS through the olfactory nerves [33], the peripheral nervous system [34, 35], or even the bloodstream [36]. Interestingly, we observed the viral NP antigen in endothelial cells of brain, which provides direct evidence that the HPAIV H5N1 likely invades the CNS by replicating in blood vessels in the brain, and contributes to the development of severe nervous symptoms. Based on our evidence, we consider this to be one of the routes for HPAIV H5N1 to invade CNS. Severe CNS disorders in birds are probably one of the main causes for mortality when neurons are infected; massive edema due to virus infection-induced altered vascular permeability and multi-organ failure are commonly blamed for high mortality in HPAIV infected birds.

Mechanisms for viral penetration of the blood-brain barrier in the brain have been investigated previously. The virus may invade neurons through the opening of endothelial cell junctional complexes (para-cellular route) [37] or through vesiculo-tubular structures (trans-cellular route) [38]. It could reach the vessels in the brain through the bloodstream, or via a “Trojan horse mechanism” where viral particles are transported through infection of leukocytes and/or mononuclear cells [39]. In our study, viral RNA or antigen was easily detected in blood or sera and infected macrophages and monocytes, which suggests that the endothelial cells may play a crucial role in viral penetration of the blood-brain barrier, leading to severe necrotizing encephalopathy and death. Our finding that endothelial cells of the cerebellum were also strongly positive in viral NP antigen supports this hypothesis (Fig 4).

In this report, the viral antigen was strongly expressed in the acinar epithelium of pancreas, giving rise to the possibility of a potential role of the pancreas in viral pathogenesis. Evidence showed that the virus also replicated in lymphocytes of follicular layer of the bursa, which may be significant in inducing immunity against the virus, a key process for recovery of sick birds from infection. A remarkable finding in our study is the isolation of virus from testicular tissue samples; infection at this site resulted in embryos with severe hemorrhages and congestion (Fig 1E) leading to death within 48hrs post-infection. Moreover, high viral RNA levels were detected from testicular tissue (Fig 2), with viral NP antigen expressed in between seminephrous tubules (Fig 7E). Strikingly, the viral antigen was also detected inside these seminephrous tubules sticking to sperm. However, sexual transmission for apparently healthy cocks to spread HPAIV H5N1 during the incubation period is probably a scenario of low or unlikely probability. It is likely that sperm collected from infected cocks with healthy appearance could disseminate disease to both uninfected birds and farm handlers and workers either via natural insemination or during application of artificial insemination. Thus, the findings in this report not only provide insights on HPAI H5N1 viral pathogenicity and pathogenesis in natural infection, but also emphasize the importance of biosecurity and proper handling in poultry agriculture.
